# Association between Inflammatory Marker, Environmental Lead Exposure, and Glutathione S-Transferase Gene

**DOI:** 10.1155/2013/474963

**Published:** 2013-01-17

**Authors:** Jintana Sirivarasai, Winai Wananukul, Sming Kaojarern, Suwannee Chanprasertyothin, Nisakron Thongmung, Wipa Ratanachaiwong, Thanyachai Sura, Piyamit Sritara

**Affiliations:** ^1^Department of Medicine, Faculty of Medicine Ramathibodi Hospital, Mahidol University, Bangkok 10400, Thailand; ^2^Division of Clinical Pharmacology and Toxicology, Department of Medicine, Faculty of Medicine Ramathibodi Hospital, Mahidol University, Bangkok 10400, Thailand; ^3^Office of Research Academic and Innovation, Faculty of Medicine Ramathibodi Hospital, Mahidol University, Bangkok 10400, Thailand; ^4^Health Office, Electricity Generating Authority of Thailand, Nonthaburi 11130, Thailand

## Abstract

A number of studies suggested that lead is related to the induction of oxidative stress, and alteration of immune response. In addition, modifying these toxic effects varied partly by GST polymorphism. The objectives of this study were to assess the association between the lead-induced alteration in serum hs-CRP, with GSTM1, GSTT1, and GSTP1 Val105Ile genetic variations and the health consequence from environmental lead exposure. The 924 blood samples were analyzed for blood lead, CRP, and genotyping of three genes with real-time PCR. Means of blood lead and serum hs-CRP were 5.45 **μ**g/dL and 2.07 mg/L. Both CRP and systolic blood pressure levels were significantly higher for individuals with blood lead in quartile 4 (6.48–24.63 **μ**g/dL) compared with those in quartile 1 (1.23–3.47 **μ**g/dL, *P* < 0.01). In particular, in men with blood lead >6.47 **μ**g/dL the adjusted odds ratio (OR) of CRP levels for individuals with GSTP1 variants allele, GSTM1 null, GSTT1 null, double-null GSTM1, and GSTT1 compared with wild-type allele was 1.46 (95% CI; 1.05–2.20), 1.32 (95% CI; 1.03–1.69), 1.65 (95% CI; 1.17–2.35), and 1.98 (95% CI; 1.47–2.55), respectively. Our findings suggested that lead exposure is associated with adverse changes in inflammatory marker and SBP. GST polymorphisms are among the genetic determinants related to lead-induced inflammatory response.

## 1. Introduction

Among the deleterious impacts of environmental toxicants on human health, lead exposure may be more hazardous, with various effects and public health concerned. Previous studies suggested that lead was known to induce oxidative stress by increasing the production of reactive oxygen species (ROS), and antioxidants have an important role to ameliorate lead toxicity [1, 2]. Free radicals can lead to cell membrane damage, via lipid peroxidation, which in turn triggers the signaling cascades of inflammatory process. Furthermore, inflammation is thought to act as a mediator of the adverse health effects caused by lead exposure. Kim et al. [3] found a significant association between indicators of inflammation (higher serum TNF-*α* and WBC count) in male subjects with blood lead ≥2.51 *μ*g/dL. Moreover, high-sensitivity C-reactive protein (hs-CRP), one of inflammatory markers, has been reported with lead exposure. The lead-exposed workers had significantly high hs-CRP level (4.49 mg/L) compared to controls (0.99 mg/L) and blood lead showed a significant positive correlation with serum hs-CRP (*r* = 0.75, *P* < 0.01) [4].

The glutathione S-transferase (GSTs) is known as phase II xenobiotic-metabolizing enzyme that plays an important role in the detoxifying of various toxicants, and toxic intermediates produced by each biotransformation pathway, including ROS. Intensified production of ROS caused by lead has been suggested to promote the downregulation of glutathione (GSH) which acts as scavenging molecule for oxidants and toxic electrophiles [5, 6]. Since GST protein catalyzes the conjugation of electrophile to GSH, therefore alteration in this protein both in expression and activity might affect the individual response to the oxidative damage or inflammation resulted by lead exposure. Genetic variations in GSTs gene (i.e., GSTM1, GSTT1, and GSTP1) contribute to interindividual differences in response to chemical and carcinogenic compounds. The null genotype of GSTM1 or GSTT1 resulted in the absence of enzyme activity and GSTP1 Ile105Val lead to decrease this enzyme activity. A combined mutation in these genes can modify the potential risks caused by toxic substance itself or products from stages of oxidative stress and inflammation. In particular, one study investigated whether lead exposure and genetic polymorphisms of GSTM1 and TNF-*α* on change in inflammatory markers in nonoccupationally exposed adults [3]. The result showed an effect of blood lead on the TNF-*α*, IL-6, and WBC only in individuals with the GSTM1 null genotype. Songdej et al. [7] also revealed that the odds of CRP level in men with blood lead level ≥ 3.09 *μ*g/dL were 77% greater as compared to those with a blood lead level ≤1.16 *μ*g/dL. 

Nowadays, lead also is a cumulative heavy metal and its distribution in environment continues to be a matter of health concern by various routes of exposure in general population (i.e., contaminated food and beverage, and air pollution). Moreover, immunotoxicology studies that dealt with indirect effect on the immune system are of increasing attention, especially a number of agents like inhibitors of immune function (such as metals; lead, cadmium, mercury, and arsenic). The relation between blood lead and CRP was not well documented and currently there is unclear whether the risk related to low level of lead in exposed population modulated by variants in the GSTs gene was associated with inflammation marker. The objectives of this study were to assess the association between the lead-induced alteration in an inflammatory marker, hs-CRP, with the GSTM1, GSTT1 and GSTP1 genetic variations and the potential health consequence from environmental lead exposure. 

## 2. Materials and Methods

### 2.1. Study Subjects

The Electric Generating Authority of Thailand (EGAT) Study was the first cohort study of chronic disease in Thailand, originally designed in 1985 (known as EGAT 1), and mainly covered multidisciplinary researches related to cardiovascular disease (CVD) risk factors such as nutrition and toxicology. The 924 male subjects in this study (in 2009 known as the third survey of EGAT 2) completed a self-administered questionnaire, underwent a physical examination, and provided fasting blood samples [8]. The study was approved by the Committee on Human rights Related to Researches Involving Human Subjects, Faculty of Medicine Ramathibodi Hospital, Mahidol University, in accordance with the ethical standards laid down in the 1964 Declaration of Helsinki. All participants gave their informed consent prior to their inclusion in the study. 

Blood samples were obtained by venipuncture after an overnight fast and immediately centrifuged at 2000 rpm for 15 min and stored at −80°C until analysis, except whole blood (for lead measurement and genotyping assay).

### 2.2. Determination of Lead in Blood

Whole blood lead concentrations were measured by graphite furnace atomic absorption spectrometry (GFAAS) with Zeeman background correction after dilution of the blood (1 : 10) with Triton X-100 solution containing diammonium hydrogen phosphate and nitric acid [9]. The concentration was expressed as micrograms per deciliter.

### 2.3. Determination of Serum hs-CRP

 Automated hs-CRP measurements were performed with immunonephelometry (Beckman Coulter, Milano, Italy), following the manufacture's instruction, and using reagents and calibrators specifically designed for high sensitivity measurement. The detection limit was 0.2 mg/L [10]. 

### 2.4. Genotype Analyses

The genomic DNA was extracted from the lymphocytes by a modified salting out procedure [11] and frozen at −20°C until analysis. The genetic polymorphisms of *GSTM1*, *GSTT1*, and *GSTP1 *were performed by real-time polymerase chain reaction (real-time PCR) according to the method of TaqMan SNP Genotyping Assays on an ABI 7500 instrument (Applied Biosystems, Foster City, CA, USA), in 96-well format. The TaqMan Assay included the forward target-specific polymerase chain reaction (PCR) primer, the reverse primer, and the TaqMan MGB probes labeled with 2 special dyes: FAM and VIC. The concentrations of probes were 0.04 *μ*M. Amplification of 20 ng of DNA was performed during 40 cycles in a reaction volume of 10 *μ*L. TaqMan Universal PCR Master Mix was used for analysis. Thermocycling conditions were 95°C for 15 seconds, follow by 60°C for 1 minute. Information of specific probe and primers is available on the National Cancer Institute's SNP500 database web page at http://variantgps.nci.nih.gov/cgfseq/pages/snp500.do [12].

### 2.5. Statistical Analysis

Statistical analyses were carried out using the SPSS 16.0 for window software (SPSS, Inc., Chicaco, IL, USA). Lead values were expressed as mean ± SD. The comparisons between variables were examined by the Student's *t*-test and analysis of variance (ANOVA). Genotype distribution was analyzed with *χ* ^2^. A *P* value of 0.05 was used as the criterion for statistical significance. Quartile cutoffs of blood lead based on the weighted distribution in the study samples were Q1; 1.23–3.47 *μ*g/dL, Q2; 3.48–4.55 *μ*g/dL, Q3; 4.56–6.47 *μ*g/dL, Q4; 6.48–24.63 *μ*g/dL. To investigate associations between blood lead, genetic variations of GSTs gene, and inflammatory markers, adjusted logistic regression model was used for further analysis.

## 3. Results

Characteristics of the study participants and distribution of variables classified by quartiles of blood lead levels are presented in Table 1. Mean of blood lead in all subjects was 5.45 *μ*g/dL (range 1.23–24.63 *μ*g/dL). Age, BMI, and diastolic blood pressure showed no differences with respect to blood lead levels. Smoking cigarette showed significant effects on blood lead levels (9.29 *μ*g/dL for current smokers versus 4.93 *μ*g/dL for nonsmokers, *P* < 0.01), whereas alcohol consumption demonstrated no significant results. Moreover, our findings revealed that in the fourth quartile, blood lead levels in current smokers were significantly higher than in non-smokers (12.34 versus 8.76 *μ*g/dL, resp., *P* < 0.01). The inflammatory marker, hs-CRP level, increased with the elevated blood lead level (Q1–4 of blood lead with hs-CRP 1.54, 1.87, 2.79, and 4.12 mg/L, resp.) with statistical differences between quartile 1, and 4 (*P* < 0.01). Similar to CRP, the means of systolic blood pressure in subjects with the highest quartile and the lowest quartile of blood lead were statistical differences (132.1 mmHg for Q4 and 114.8 mmHg for Q1, *P* < 0.01).

Genotype frequencies of GSTM1, GSTT1, and GSTP1 are demonstrated in Table 2. The percentages of GSTM1 and GSTT1 null genotypes were 47.6 and 69.1, respectively. The combined genetic variations of GSTM1 and GSTT1 were presented as double-null genotype with 34.1%, null/present 48.5% and double present 17.4%. For GSTP1 Ile105Val, the genotype distribution was done according to the Hardy-Weinberg equilibrium (*P* = 0.21). However, we also classified the two groups of GSTP1 as wild type (Ile/Ile genotype, 55.9%) and variant allele (Ile/Val and Val/Val genotype, 44.1%) for further statistical analysis.

Table 3 illustrates the genetic impact of GSTs gene on the blood lead (only in the quartile 4) and CRP. The OR of serum hs-CRP for subjects with at least one variant allele of GSTP1 was 1.46 (95% CI: 1.05–2.20). For gene deletion of GSTM1 and GSTT1, the adjusted ORs (95% CI) of CRP were 1.32 (1.03–1.69) for null GSTM1 and 1.65 (1.17–2.35) for null GSTT1. In order to focus on modifying effects of the combined genotypes on lead exposure and inflammatory response, we analyzed the two polymorphisms together and divided the data set into 3 groups (GSTM1/GSTT1 double −/−; GSTM1/GSTT1 −/+ and +/−; GSTM1/GSTT1 double +/+). Also, subjects with the double-null genotypes of GSTM1 and GSTM1 showed higher ORs (1.98, CI: 1.47–1.31) than those with GSTM1/GSTT1 −/+ and +/− (1.07, CI: 0.88–1.31).

## 4. Discussion

 Lead is toxic to various organ systems, even at low level of exposure. To date, there are some pieces of evidence suggest that lead exposure increases the production of oxidative stress and inflammatory response, as seen by elevation of blood malondialdehyde (MDA) and proinflammatory cytokines [4, 13]. Therefore, environmental lead-exposed people were more susceptible to face with threats to human health. Khan et al., [4] also suggested that one of the mechanisms of lead toxicities related to a vicious cycle is formed by oxidative stress and inflammation. In this study, we have measured blood lead level and an inflammatory marker (hs-CRP) together with genotyping study (GSTs polymorphism). 

 Mean of blood lead level in this study population was 5.45 *μ*g/dL (*n* = 924, aged 35–60 years) which was higher than the geometric mean (1.89 *μ*g/dL; *n* = 6497, aged 40–79 years) of US population from NHANES III project (The National Health and Nutrition Examination Survey III; 1999–2004) [7]. Smoking status and systolic blood pressure showed statistical differences with respect to blood lead level (Table 1) and similar to other studies. Falq et al. [14] reported that blood lead levels were significantly increased with age, smoking status, and alcohol consumption. A significant source and mechanism of observed lead exposure by smoking cigarette could be explained with the promoting transportation of airborne lead into the respiratory tract by smoke particles [15]. Absorption rate varied widely depending on factors such as smoking intensity and depth of inhalation. In addition, lead found in tobacco comes from atmospheric pollution by which lead was trapped on the surface of the leaf and around 11% of the lead in cigarette entered the smoke that was emitted from the cigarette [16]. 

CRP is one of the important markers of inflammation. The primary regulators of CRP are the cytokines interleukin (IL)-6 and IL-1*β* and tumour necrosis factor (TNF)-*α*. Mean of CRP level in our nonoccupational lead participants was 2.07 mg/L which was higher than the finding of Khan et al. (0.995 mg/L, *n* = 61) [4]. Previous studies reported the effect of lead exposure on immune response of experimental animals and human both in immune-stimulating and immune-suppression effects [17, 18]. In the present study, blood lead level was associated with the elevated blood CRP (Table 1) which could be supported by another study with markedly elevated lead that produces oxidative stress and acted on various pathways through specific mediators such as Il-6, TNF-*α* that amplified CRP production from liver [4]. Moreover, one investigation in Nigerian lead-exposed workers found a decreased immune status and significant raised CRP (*P* < 0.01) in response to elevated blood lead level [19]. Another finding in this study was a relationship between systolic blood pressure and blood lead level. Animal study indicated that chronic lead exposure could stimulate a cascade of events including oxidative stress and inflammation that might result in the progression of hypertension [20]. In addition, basic research suggested that inflammation might contribute to a rise in blood pressure by promoting changes in the endothelium, which lined the walls of the blood vessels. Elevated levels of CRP can induce structural and functional changes in the endothelium ultimately contributing to the rise in blood pressure [21].

 Since lead exposure and CRP were known to be involved in oxidative stress and this event also contributed to a major role of GSTs gene in antioxidant defense mechanism. GSTM1, GSTT1, and GSTP1 genes are polymorphic in humans which result in interindividual differences in lead toxicity. Genotyping results of three genes in this study population are shown in Table 2. We observed that the genotype frequencies of GSTM1 and GSTT1 deletions in this Thai population were 47.6% and 69.1%, respectively. For GSTM1/GSTT1 genotype combination, the frequency of GSTM1/GSTT1double-null genotype was 34.1%. These results were similar to the report of the Chinese population [22]. Moreover, our results also indicated the uncommon homozygous GSTP1 −105Val/Val in Thai (5.8% from this study and 5.1% from our previous study; Khansakorn et al., [23]), Korean (5.2%) [24], and Chinese (3.5%) [25] which were lower than those reported in Austrian (10.5%) [26]. 

Gene deletions of GSTT1/GSTM1 and a missense mutation at codon 105 of GSTP1 exhibited alterations in enzyme activity. These changes indicated increasing detrimental function for scavenging free radicals or toxicants in the human body, including lead. The influences of blood lead on inflammation, particularly level > 6.47 *μ*g/dL, were found in individuals with the GSTM1, GSTT1 null genotype, and GSTP1 variant allele. Those men appeared to be more sensitive to lead exposure with the risks of elevated inflammation levels. The changes in GST enzyme activity may be reduced in Pb-GSH conjugates and excretion, leading to accumulation of lead in blood. In turn, previous studies also reported that lead could influence on suppression of T-helper type 1 cells and enhancement of T helper type 2 (Th 2) cells [27, 28]. In addition, these Th2 cells are significantly related to IL-6 production which played a role in the upregulation of both CRP and fibrinogen [29, 30]. Findings from this study and from Kim et al. [3] suggested that GSTM1, GSTT1, and GSTP1 polymorphisms were genetic factors associated with the lead-induced inflammatory response which indicated the existence of susceptible lead-exposed individuals even at low environmental concentration. 

However, this study presented some limitations in considering the influence of mixed chemicals in the environment, the lack of direct GST enzyme activity measurement indicating the association of genetic background with altered catalytic activity, and uncontrolled other factors modulating cytokine production. The potential important strength of this study was the large dataset with a representative of the general population of Thailand. In addition, biomarker of exposure as blood lead and inflammatory marker as serum hs-CRP were firstly determined concurrently with biomarker of susceptibility as GSTs polymorphism among Thai ethnic. These findings could indicate a consistent association between blood lead level and inflammation and also elucidate the biological mechanism behind possible genetic background differences.

In conclusion, environmental lead exposure, monitored by blood lead levels, affects inflammatory marker and increasing of both markers may lead to adverse change in blood pressure. Genetic polymorphisms of GSTs are associated with lead-induced inflammatory response.

## Figures and Tables

**Table 1:** Means of blood lead levels and other variables classified by 4 leadquartiles among men participating in the EGAT Study project.

Characteristic		Blood lead Quartiles			
	Total	Quartile 1	Quartile 2	Quartile 3	Quartile 4
	(*N* = 924)	(*N* = 218)	(*N* = 222)	(*N* = 242)	(*N* = 242)
Blood lead level, mean (rang), *μ*g/dL	5.45	2.44	3.95	5.77	9.21
	(1.23–24.63)	(1.23–3.47)	(3.48–4.55)	(4.56–6.47)	(6.48–24.63)
Age, mean (SD), years	42.55 (3.15)	42.94 (6.33)	42.17 (5.29)	42.33 (5.29)	42.78 (7.30)
Body mass index, mean (SD), kg/m^2^	23.99 (6.11)	24.59 (3.25)	23.56 (6.21)	23.78 (9.19)	24.06 (6.24)
Mean blood lead (SD), *μ*g/dL; classified by smoking status					
Nonsmokers	4.93 (2.36)	2.09 (0.96)	3.79 (0.88)	4.90 (1.01)	8.76 (1.12)
Former smokers	6.07 (2.94)	2.45 (0.75)	3.68 (1.23)	4.81 (0.97)	9.23 (1.39)
Current smokers	9.29^a^ (4.26)	2.81 (1.21)	4.08 (1.08)	5.43 (1.14)	12.34^ a,b^ (5.32)
Mean blood lead (SD), *μ*g/dL; classified by alcohol consumption,					
Nondrinkers	5.32 (2.36)	2.12 (0.96)	3.56 (1.12)	4.84 (1.24)	7.96 (3.12)
Lightdrinkers	4.96 (1.98)	1.99 (0.35)	3.78 (1.04)	5.12 (1.98)	8.82 (3.07)
Ex-drinkers	5.17 (2.18)	2.32 (0.74)	3.44 (1.31)	4.97 (1.69)	8.23 (2.98)
Current drinkers	6.49 (4.99)	2.41 (1.01)	4.17 (1.36)	5.33 (1.54)	11.07 (5.31)
Serum hs-CRP level, mean (SD), mg/L	2.07 (1.62)	1.54 (0.79)	1.87 (0.96)	2.79 (1.36)	4.12^c,d^ (2.18)
Systolic BP, mean (SD), mmHg	124.4 (10.55)	114.8 (6.09)	123.7 (8.06)	126.8 (10.14)	132.1^c ^(16.13)
Diastolic BP, mean (SD), mmHg	77.29 (15.38)	77.44 (8.73)	76.78 (11.75)	77.62 (16.83)	77.31 (10.77)

^a,b ^Significantly different from never smoked and former smoker, respectively, *P* < 0.01.

^c,d ^Significantly different from blood lead quartiles 1 and 2, respectively *P* < 0.01.

**Table 2:** Genotype frequencies for GSTP1, GSTM1, and GSTT1 (*N* = 924).

Gene	Variation	Genotype	Frequency	
			Number	Percentage
GSTP1 (rs1695)	Ile105Val	Ile/Ile	517	55.9
		Ile/Val and Val/Val	407	44.1
GSTM1	Deletion	+/+	484	52.4
		−/−	440	47.6
GSTT1	Deletion	+/+	286	30.9
		−/−	638	69.1
GSTM1 and GSTT1	Deletion	+/+	161	17.4
		−/+ or +/−	448	48.5
		−/−	315	34.1

**Table 3:** The odds ratio (OR) for increasing inflammatory mark by genetic variations of GSTs in relation to blood lead level >6.47 *μ*g/dL.

GST genetic variation	CRP*	
	OR	95% CI
GSTP1 (Ile105Val)		
Ile/Ile	1	Reference
Ile/Val and Val/Val	1.46	1.05−2.20
GSTM1		
+/+	1	Reference
−/−	1.32	1.03−1.69
GSTT1		
+/+	1	Reference
−/−	1.65	1.17−2.35
GSTM1 and GSTT1		
+/+	1	Reference
+/− or −/+	1.07	0.88−1.31
−/−	1.98	1.47−2.55

CRP: C-reactive protein; OR: odds ratio; CI: confidence interval.

*With adjustment for age, body mass index, smoking status, alcohol use, and blood pressure.
